# Incidence and mortality of kidney cancers, and human development index in Asia; a matter of concern

**DOI:** 10.15171/jnp.2017.06

**Published:** 2016-08-07

**Authors:** Masoumeh Arabsalmani, Abdollah Mohammadian-Hafshejani, Mahshid Ghoncheh, Fatemeh Hadadian, Farhad Towhidi, Kamran Vafaee, Hamid Salehiniya

**Affiliations:** ^1^Department of Epidemiology and Biostatistics, School of Public Health, Zahedan University of Medical Sciences, Zahedan, Iran; ^2^Social Determinants in Health Promotion Research Center, Hormozgan University of Medical Sciences, Bandar Abbas, Iran; ^3^Department of Epidemiology and Biostatistics, School of public health, Hamadan University of Medical Sciences, Hamadan, Iran; ^4^Department of Medical Surgical Nursing, School of Nursing and Midwifery, Kermanshah University of Medical Sciences, Kermanshah, Iran; ^5^Imam Reza Hospital, Kermanshah University of Medical Sciences, Kermanshah, Iran; ^6^Zabol University of Medical Sciences, Zabol, Iran; ^7^Department of Epidemiology and Biostatistics, school of public health, Tehran University of Medical Sciences, Tehran, Iran

**Keywords:** Kidney cancer, Human development index, Incidence, Mortality, Asia

## Abstract

**Background:**

The incidence and mortality of kidney cancer have steadily increased by 2%- 3% per decade worldwide, and an increased risk of kidney cancer has been observed in many Asian countries. The information on the incidence and mortality of a disease and its distribution is essential for better planning for prevention and further studies.

**Objectives:**

This study aimed to assess the incidence and mortality of kidney cancer and their correlation with the human development index (HDI) in Asia.

**Materials and Methods:**

This ecological study was based on GLOBOCAN data Asia for assessment the correlation between age-specific incidence rate (ASIR) and age-specific mortality rate (ASMR) with HDI and its details that include life expectancy at birth, mean years of schooling and gross national income (GNI) per capita. We use of correlation bivariate method for assessment the correlation between ASIR and ASMR with HDI and its components.

**Results:**

A total of 121 099 kidney cancer cases were recorded in Asian countries in 2012.Overall, 80 080 cases (66.12%) were males. Sex ratio was 1.95. The three countries with the highest number of new patients were china (66 466 cases), Japan (16 830 cases), India(9658 cases), respectively. Positive correlation were seen between HDI and ASIR of kidney cancer 0.655 (*P* = 0.001), and HDI and ASMR of kidney cancer 0.285 (*P* = 0.055).

**Conclusions:**

A positive relationship between ASIR and the HDI was seen. The relationship is due to risk factors in countries with high development such as older age, smoking, hypertension, obesity, and diet. However, ASMR showed no significant relationship with HDI.

**Implication for health policy/practice/research/medical education:**

The result of this article can help policy-makers and health managers to find the cause of the incidence and mortality of kidney cancer in Asia. This study provides information on the incidence and mortality of the cancer and its distribution in terms of geographical areas, so it provides information for better planning for prevention and further studies. Increasing public awareness of the cancer risk factors is a high priority in low development country.

## 1. Background

It was estimated that 14.9 million incidence cases of cancer and 8.2 million deaths from cancer with 196.3 million disability-adjusted life years (DALYs) in 2013(1). Kidney cancer was the 13th most common cancer worldwide in both sexes (2,3), and the 9th and the 14th type of cancer in men and women, respectively, and the 16th cause of death from the disease in 2012 (4). In the world, age-specific incidence rate (ASIR) of kidney cancer was 4.4, mortality of kidney cancer was 1.8, and its 5-year prevalence was 17.5 in 2013 (1).

A difference is observed in the incidence of kidney cancer by 15 times around the world (5). Therefore, the highest incidence and mortality rates are attributable to Europe, North America, and Australia, while the lowest to Asia and Africa (2,6-8). In 2006, there were approximately 209000 new cases and 102000 deaths from renal cell carcinoma (RCC) worldwide, of which 39000 new cases and 13000 deaths occurred in the United States (9-12). In 2002, the incidence rates in Asian countries such as Japan and Republic Korea were 2509 and 481 cases per 100000, respectively, while 8567 and 901 cases were seen in Canada and United States, respectively (13). In 2012, the incidence rates per 100000 person-years in nine Asian countries were 4.6 in men and 3.1 in women, respectively. Given that the incidence and mortality have steadily increased by 2%-3% per decade worldwide (10,14-24), reduction of mortality has been reported from many developed European countries since the 1990s (19,20).

An increased risk of kidney cancer was observed in many Asian countries such as Korea, China, Hong Kong, Singapore, and Japan (25). A significant increase in the incidence and mortality rates in Asian countries occurred along with remarkable changes in food supply and diet (26). It appears that lifestyle plays an important role in the development of RCC. However, the cause of the differences cannot be understood, it is assumed that different trends are related to early detection, improved access to health care, complex diagnostic imaging, and treatment availability (27,28).

According to studies conducted in Asian countries, a number of common risk factors for RCC include sex, hypertension, diabetes mellitus, high body mass index (BMI), a medical history of kidney disease, smoking, low physical activity, and Western diet (25,29,30), all of which were related to India (30), Japan (29) and Malaysia (31). Other environmental, genetic and hormonal factors were studied, but no definitive conclusions. The cancer primarily affects men and women in the fifth and sixth decades of their life, and one of the specific characteristics of the disease is asymptomatic (19,20,32).

Deciding factors for diagnosis and treatment of patients with locally and advanced RCC are sex, race, the income level and social economic status, which influence the provider and the recipient of health care and decision-making on the disease (33). Studies showed that the incidence of kidney cancer is affected by economic and social disparities (18,19). It reflects the regional disparities in human development. To study the trend and its relationship with risk factors, the human development index (HDI) is useful. The index to classify cancer for globalization is beneficial because it considers education, life expectancy and the national income (34-37). The assumption is that the populations of developing countries have higher mortality rates than the incidence compared with developed countries (38,39). After several decades of increasing trend in the incidence and mortality of kidney cancer, rates are stable, or are started to decrease in many Western countries (40). It seems it is due to reducing the prevalence of tobacco consumption, and improving health professional than decades ago because the prevalence of smoking is higher among people who live below the poverty line (31.5%) than those at the top of this level (19.6%) (7,40).

## 2. Objectives

The information on the incidence and mortality of a disease and its distribution in terms of geographical areas is essential for better planning for prevention and further studies. There is, probably, a relationship between the development and cancer incidence and mortality. Considering lack of a study to investigate kidney cancer incidence and deaths in Asia, this study aimed to assess the incidence and mortality of kidney cancer and their correlation with the HDI in Asia.

## 3. Materials and Methods

This study was an ecologic study in Asia for assessment of the correlation between age-specific incidence rate (ASIR) and age-specific mortality rate (ASMR) with HDI and its details that include mean years of schooling, life expectancy at birth and gross national income (GNI) per capita. Data about the ASIR and ASMR for every Asian country for the year 2012 get from global cancer project (http://globocan.iarc.fr/Default.aspx) (41). HDI extracted from Human Development Report 2013 included information about HDI and its details for every country in the word for year 2012 (42).

Method of estimation of the age-specific incidence and mortality rates in the global cancer project by international agency for research on cancer is as follows:

### 3.1. Age-specific incidence rate

The methods of estimation are country specific and the quality of the estimation depends upon the quality and on the amount of the information available for each country. In theory, there are as many methods as countries, and because of the variety and the complexity of these methods, an overall quality score for the incidence and mortality estimates combined is almost impossible to establish. However, an alphanumeric scoring system, which independently describes the availability of incidence and mortality data, has been established at the country level. The combined score is presented together with the estimates for each country with an aim of providing a broad indication of the robustness of the estimation. More details about the GLOBOCAN project were previously published (4,43,44).

### 3.2. Age-specific mortality rate

Depending on the degree of detail and accuracy of the national mortality data, six methods have been utilized in the following order of priority: 1-Rates projected to 2012 (69 countries), 2) Most recent rates applied to 2012 population (26 countries), 3) Estimated as the weighted average of regional rates (1 country), 4) Estimated from national incidence estimates by modelling, using country-specific survival (2 countries), 5) Estimated from national incidence estimates using modelled survival (83 countries), 6) The rates are those of neighboring countries or registries in the same area (3 countries( (4,44,45).

### 3.3. Human development index

HDI, a composite measurement of indicators along three dimensions: life expectancy, educational attainment and command over the resources needed for a decent living. All groups and regions have seen notable improvement in all HDI components, with faster progress in low and medium HDI countries. On this basis, the world is becoming less unequal. Nevertheless, national averages hide large variations in human experience. Wide disparities remain within countries of both the North and the South, and income inequality within and between many countries has been rising (42).

### 3.4. Ethical issues

The research followed the tenets of the Declaration of Helsinki.

### 3.5. Statistical analysis

In this study, we use correlation of coefficient for assessment of the correlation between ASIR and ASMR with HDI and its details. All reported *P* values are two-sided, and statistical significance was assumed if *P*<0.05. Statistical analyses were performed using SPSS (version 15.0, SPSS Inc.).

## 4. Results

A total of 121099 kidney cancer cases were recorded in Asian countries in 2012. Overall, 80080 cases (66.12%) were males and 41019 cases (33.87%) females. Sex ratio in Asia was 1.95. The five countries with the highest number of new patients were china (66466 cases), Japan (16830 cases), India (9658 cases), Republic Korea (5651 cases), and Turkey (3992 cases), respectively.

**Figure 1:**
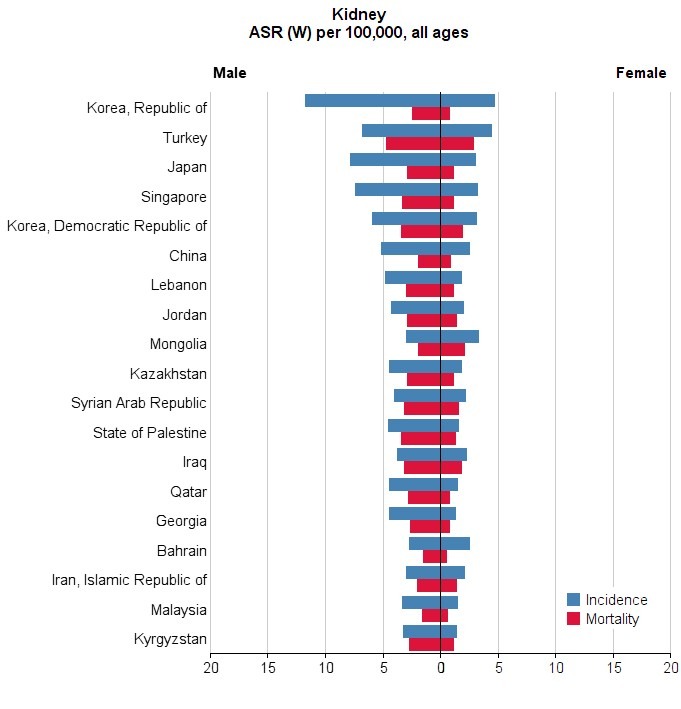
ASIR and ASMR from kidney cancer in Asia in 2012.

**Figure 2:**
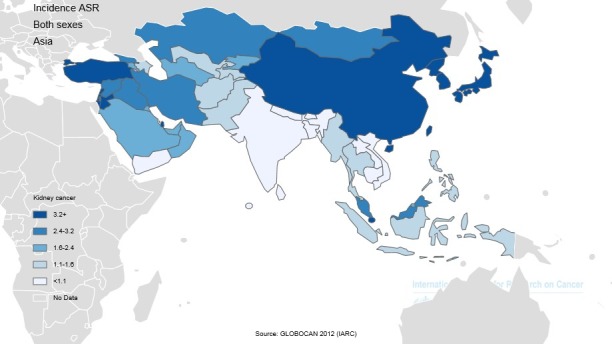
Distribution of the ASIR of kidney cancer in Asia in 2012

Among Asian countries, five countries with the highest standardized incidence rates of the cancer were Republic Korea with 8 per 100000, Turkey with 5.6 per 100000, Japan with 5.3 per 100000, Singapore with 5.2 per 100000, and Korea, Democratic Republic of 4.3 per 100000, respectively. Five countries with the lowest standardized incidence rates of the cancer were Maldives with 0 per 100000, Bhutan with 0.6 per 100000, Yemen with 0.6 per 100000, Bangladesh with 0.8 per 100000, and Sri Lanka with 0.9 per 100000, respectively. The number, crude and standardized incidence rates of the cancer in Asian countries based on sex are presented in Table 1. The countries with the highest and lowest ASIR in both sexes are observable in Table 1, Figure 1 and Figure 2.

**Table 1:** Proportion, crude, and ASIR of kidney cancer in Asian countries in 2012

**Kidney - Estimated incidence, all ages: both sexes**				**Kidney - Estimated incidence, all ages: male**				**Kidney - Estimated incidence, all ages: female**			
**Population**	**Number**	**Crude rate**	**ASR (W)**	**Population**	**Number**	**Crude rate**	**ASR (W)**	**Population**	**Numbers**	**Crude rate**	**ASR (W)**
Korea, Republic of	5651	11.6	8.0	Korea, Republic of	1763	7.2	4.7	Korea, Republic of	3888	16.1	11.7
Turkey	3992	5.4	5.6	Turkey	1656	4.4	4.4	Japan	11141	18.1	7.8
Japan	16830	13.3	5.3	Mongolia	37	2.6	3.3	Singapore	272	10.3	7.4
Singapore	401	7.6	5.2	Singapore	129	4.9	3.2	Turkey	2336	6.3	6.8
Korea, Democratic Republic of	1318	5.4	4.3	Korea, Democratic Republic of	566	4.5	3.1	Korea, Democratic Republic of	752	6.2	5.9
China	66466	4.9	3.8	Japan	5689	8.8	3.0	China	44372	6.3	5.1
Qatar	33	1.7	3.5	Bahrain	9	1.8	2.5	Lebanon	100	4.8	4.8
Jordan	129	2.0	3.2	China	22094	3.4	2.5	State of Palestine	49	2.3	4.5
Lebanon	142	3.3	3.2	Iraq	262	1.6	2.3	Kazakhstan	314	4.0	4.4
Mongolia	66	2.3	3.1	Syrian Arab Republic	171	1.6	2.2	Qatar	27	1.8	4.4
Syrian Arab Republic	467	2.2	3.1	Iran	660	1.8	2.1	Georgia	123	6.1	4.4
State of Palestine	71	1.7	3.1	Oman	15	1.3	2.1	Jordan	87	2.6	4.3
Kazakhstan	491	3.0	2.9	Jordan	42	1.3	2.0	Syrian Arab Republic	296	2.8	4.0
Iraq	581	1.7	2.9	Saudi Arabia	166	1.3	1.8	Iraq	319	1.9	3.7
Georgia	167	3.9	2.7	Kazakhstan	177	2.1	1.8	Timor-Leste	9	1.5	3.4
Bahrain	23	1.7	2.6	Lebanon	42	1.9	1.8	Armenia	57	3.9	3.4
Iran	1641	2.2	2.6	United Arab Emirates	22	0.9	1.8	Malaysia	415	2.8	3.3
Malaysia	611	2.1	2.4	State of Palestine	22	1.0	1.6	Kyrgyzstan	57	2.1	3.2
Saudi Arabia	454	1.6	2.3	Qatar	6	1.3	1.5	Iran	981	2.6	3.0
United Arab Emirates	64	0.8	2.3	Malaysia	196	1.4	1.5	Mongolia	29	2.1	3.0
Kyrgyzstan	91	1.7	2.2	Kuwait	9	0.8	1.5	Saudi Arabia	288	1.8	2.8
Kuwait	34	1.2	2.2	Brunei	3	1.5	1.4	Bahrain	14	1.6	2.7
Timor-Leste	14	1.2	2.1	Kyrgyzstan	34	1.2	1.4	United Arab Emirates	42	0.7	2.6
Oman	36	1.2	2.1	Georgia	44	1.9	1.3	Kuwait	25	1.5	2.6
Armenia	78	2.5	1.9	Turkmenistan	26	1.0	1.2	Turkmenistan	49	1.9	2.5
Turkmenistan	75	1.5	1.8	Indonesia	1132	0.9	1.0	Oman	21	1.2	2.1
Brunei	6	1.5	1.8	Philippines	367	0.8	1.0	Indonesia	2093	1.7	2.0
Indonesia	3225	1.3	1.5	Timor-Leste	5	0.9	1.0	Azerbaijan	96	2.1	2.0
Philippines	1008	1.0	1.4	Lao PDR	24	0.8	0.9	Philippines	641	1.3	2.0
Tajikistan	63	0.9	1.4	Uzbekistan	107	0.8	0.9	Tajikistan	42	1.2	2.0
Afghanistan	237	0.7	1.3	Pakistan	575	0.6	0.9	Brunei	3	1.4	1.9
Azerbaijan	135	1.4	1.3	Afghanistan	77	0.5	0.9	Afghanistan	160	0.9	1.8
Pakistan	1646	0.9	1.3	Tajikistan	21	0.6	0.9	Pakistan	1071	1.2	1.7
Uzbekistan	283	1.0	1.2	Myanmar	194	0.8	0.8	Uzbekistan	176	1.3	1.6
Thailand	1017	1.5	1.2	Thailand	373	1.0	0.8	Thailand	644	1.9	1.6
Myanmar	476	1.0	1.1	Bhutan	2	0.6	0.8	Nepal	155	1.0	1.6
Lao PDR	52	0.8	1.1	Armenia	21	1.3	0.8	Myanmar	282	1.2	1.4
Nepal	218	0.7	1.0	Azerbaijan	39	0.8	0.8	Cambodia	65	0.9	1.4
India	9658	0.8	0.9	Viet Nam	352	0.8	0.7	Sri Lanka	160	1.5	1.3
Cambodia	101	0.7	0.9	Cambodia	36	0.5	0.6	India	6620	1.0	1.3
Viet Nam	810	0.9	0.9	India	3038	0.5	0.6	Lao PDR	28	0.9	1.3
Sri Lanka	221	1.0	0.9	Nepal	63	0.4	0.5	Bangladesh	620	0.8	1.1
Bangladesh	900	0.6	0.8	Sri Lanka	61	0.6	0.5	Viet Nam	458	1.0	1.1
Yemen	112	0.4	0.6	Yemen	46	0.4	0.4	Yemen	66	0.5	0.8
Bhutan	3	0.4	0.6	Bangladesh	280	0.4	0.4	Bhutan	1	0.3	0.4
Maldives	0	0.0	0.0	Maldives	0	0.0	0.0	Maldives	0	0.0	0.0

**Figure 3:**
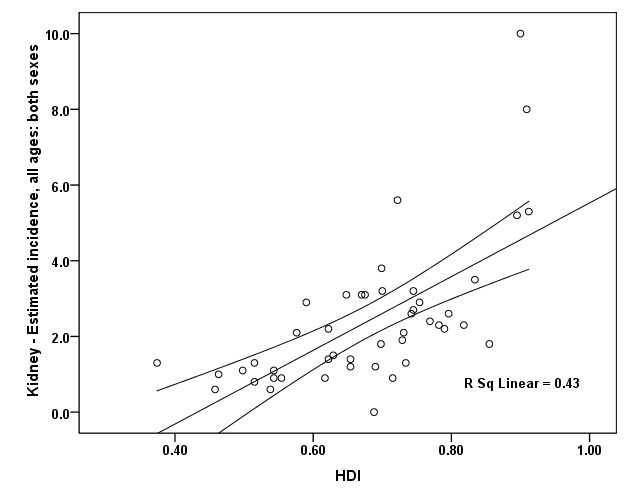
Correlation between HDI and ASIR of kidney cancer in Asia in 2012

However, in 2012, in Asia, the number of deaths due to kidney cancer was 26102 cases, 36224 cases in men and 19878 cases in women. The sex ratio (male to female) of mortality was equal to 1.82. The five countries with the highest number of deaths were china (25583 cases), Japan (8124 cases), India (5973 cases), Turkey (2656 cases), and Indonesia (2459 cases), respectively. The countries included a total of 44795 cases (89.78%) of the total mortality in Asia.

**Figure 4:**
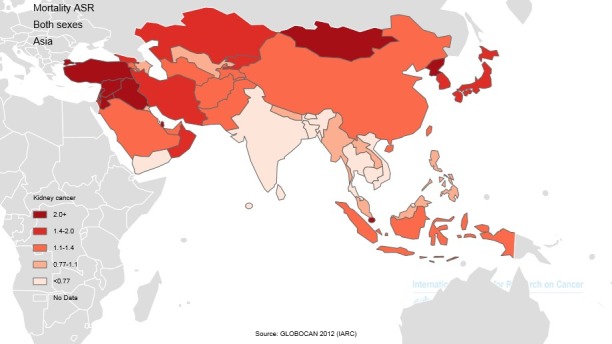
Distribution of ASMR of kidney cancer in Asia in 2012.

In Asian countries, 5 countries with the highest standardized mortality rates from the cancer were Turkey with 3.8 per 100000, Republic Korea 2.5 per 100000, Palestine with 2.4 per 100000, Syria with 2.3 per 100000, and Iraq with 2.3 per 100000, respectively. Five countries with the lowest standardized mortality rates from the cancer were Brunei with 0 per 100000, Maldives with 0 per 100000, Yemen with 0.5 per 100000, India with 0.6 per 100000, and Sri Lanka with 0.6 per 100000, respectively. The number, crude, and standardized incidence rates of the cancer in Asian countries based on sex are presented in Table 2. The countries with the highest and lowest ASIR are observable in both sexes in Table 2, Figure 1 and Figure 4.

**Table 2:** Proportion, crude, and ASMR of kidney cancer in Asian countries in 2012

**Kidney - Estimated mortality, all ages: both sexes**				**Kidney- Estimated mortality, all ages: female**				**Kidney- Estimated mortality, all ages: male**			
**Population**	**Numbers**	**CrudeRate**	**ASR(W)**	**Population**	**Number**	**Crude rate**	**ASR(W)**	**Population**	**Number**	**Crude rate**	**ASR(W)**
Turkey	2656	3.6	3.8	Turkey	1094	2.9	2.9	Turkey	1562	4.2	4.7
Korea, Democratic Republic of	794	3.2	2.5	Mongolia	21	1.5	2.1	State of Palestine	35	1.6	3.4
State of Palestine	52	1.2	2.4	Korea, Democratic Republic of	381	3.0	1.9	Korea, Democratic Republic of	413	3.4	3.4
Syrian Arab Republic	345	1.6	2.3	Iraq	206	1.2	1.8	Singapore	125	4.7	3.3
Iraq	462	1.4	2.3	Syrian Arab Republic	126	1.2	1.6	Syrian Arab Republic	219	2.1	3.1
Singapore	175	3.3	2.2	Oman	9	0.8	1.5	Iraq	256	1.5	3.1
Jordan	86	1.3	2.2	Iran, Islamic Republic of	432	1.2	1.4	Timor-Leste	8	1.3	3.1
Qatar	15	0.8	2.2	Jordan	28	0.9	1.4	Lebanon	62	3.0	3.0
Mongolia	39	1.4	2.0	State of Palestine	17	0.8	1.3	Japan	5177	8.4	2.9
Lebanon	88	2.1	2.0	Singapore	50	1.9	1.1	Jordan	58	1.7	2.9
Japan	8124	6.4	1.9	Kyrgyzstan	25	0.9	1.1	Kazakhstan	189	2.4	2.9
Timor-Leste	12	1.0	1.8	Lebanon	26	1.2	1.1	Qatar	12	0.8	2.8
Kyrgyzstan	69	1.3	1.8	Japan	2947	4.5	1.1	Kyrgyzstan	44	1.6	2.7
Kazakhstan	296	1.8	1.8	Saudi Arabia	94	0.7	1.1	Georgia	77	3.8	2.6
Iran, Islamic Republic of	1071	1.4	1.7	Kazakhstan	107	1.3	1.1	Korea, Republic of	850	3.5	2.4
Georgia	104	2.4	1.6	China	8871	1.4	0.9	Armenia	38	2.6	2.1
Korea, Republic of	1264	2.6	1.6	Qatar	3	0.6	0.8	Iran, Islamic Republic of	639	1.7	2.0
Oman	21	0.7	1.4	Korea, Republic of	414	1.7	0.8	Mongolia	18	1.3	1.9
Saudi Arabia	257	0.9	1.4	Turkmenistan	17	0.6	0.8	China	16712	2.4	1.9
China	25583	1.9	1.4	Bhutan	2	0.6	0.8	Turkmenistan	33	1.3	1.9
Turkmenistan	50	1.0	1.3	Afghanistan	68	0.4	0.8	Afghanistan	140	0.8	1.8
United Arab Emirates	25	0.3	1.3	Indonesia	866	0.7	0.8	Saudi Arabia	163	1.0	1.8
Afghanistan	208	0.6	1.3	Georgia	27	1.2	0.8	Tajikistan	32	0.9	1.7
Armenia	53	1.7	1.2	Lao PDR	19	0.6	0.8	United Arab Emirates	18	0.3	1.6
Tajikistan	49	0.7	1.2	Pakistan	483	0.5	0.8	Indonesia	1593	1.3	1.6
Indonesia	2459	1.0	1.2	Myanmar	167	0.7	0.8	Malaysia	184	1.2	1.6
Pakistan	1374	0.8	1.1	Tajikistan	17	0.5	0.7	Oman	12	0.7	1.5
Malaysia	255	0.9	1.0	United Arab Emirates	7	0.3	0.7	Bahrain	5	0.6	1.5
Myanmar	413	0.8	1.0	Timor-Leste	4	0.7	0.7	Azerbaijan	66	1.4	1.5
Kuwait	14	0.5	1.0	Uzbekistan	75	0.5	0.7	Pakistan	891	1.0	1.4
Uzbekistan	205	0.7	1.0	Philippines	221	0.5	0.6	Nepal	133	0.9	1.4
Azerbaijan	92	1.0	1.0	Viet Nam	274	0.6	0.6	Uzbekistan	130	0.9	1.3
Bahrain	7	0.5	1.0	Kuwait	3	0.3	0.6	Myanmar	246	1.0	1.3
Philippines	600	0.6	0.9	Malaysia	71	0.5	0.6	Philippines	379	0.8	1.3
Nepal	187	0.6	0.9	Azerbaijan	26	0.5	0.5	Kuwait	11	0.6	1.2
Lao PDR	40	0.6	0.9	Armenia	15	0.9	0.5	Cambodia	52	0.7	1.2
Cambodia	80	0.6	0.7	Bahrain	2	0.4	0.5	Lao PDR	21	0.7	1.0
Viet Nam	630	0.7	0.7	Thailand	233	0.7	0.5	Thailand	399	1.2	1.0
Thailand	632	0.9	0.7	Cambodia	28	0.4	0.5	Bangladesh	513	0.7	0.9
Bangladesh	776	0.5	0.7	Nepal	54	0.3	0.4	Viet Nam	356	0.8	0.9
Bhutan	3	0.4	0.6	Bangladesh	263	0.3	0.4	Sri Lanka	108	1.0	0.9
Sri Lanka	150	0.7	0.6	Yemen	40	0.3	0.4	India	4054	0.6	0.8
India	5973	0.5	0.6	India	1919	0.3	0.3	Yemen	57	0.4	0.7
Yemen	97	0.4	0.5	Sri Lanka	42	0.4	0.3	Bhutan	1	0.3	0.4
Maldives	0	0.0	0.0	Brunei	0	0.0	0.0	Brunei	0	0.0	0.0
Brunei	0	0.0	0.0	Maldives	0	0.0	0.0	Maldives	0	0.0	0.0

In Table 3, amounts related to HDI and its components for each of the Asian countries (sorted based on HDI) is shown. Accordingly, Asian countries are classified according to HDI as follows: three countries in the very high category, four countries in high, 35 countries in the middle category, three countries in low, and one in the unknown category.

**Table 3:** HDI in Asian countries in 2012

**HDI status**	**Population**	**HDI**	**Life expectancy at birth**	**Mean Year of schooling**	** GNI per capita**
Very high	Japan	0.912	83.6	11.6	32545
	Korea, Republic of	0.909	80.7	11.6	28231
High	Singapore	0.895	81.2	10.1	52613
	Brunei	0.855	78.1	8.6	45690
	Qatar	0.834	78.5	7.3	87478
	United Arab Emirates	0.818	76.7	8.9	42716
Average	Bahrain	0.796	75.2	9.4	19154
	Kuwait	0.79	74.7	6.1	52793
	Saudi Arabia	0.782	74.1	7.8	22616
	Malaysia	0.769	74.5	9.5	13676
	Kazakhstan	0.754	67.4	10.4	10451
	Georgia	0.745	73.9	12.1	5005
	Lebanon	0.745	72.8	7.9	12364
	Iran	0.742	73.2	7.8	10695
	Azerbaijan	0.734	70.9	11.2	8153
	Oman	0.731	73.2	5.5	24092
	Armenia	0.729	74.4	10.8	5540
	Turkey	0.722	74.2	6.5	13710
	Sri Lanka	0.715	75.1	9.3	5170
	Jordan	0.7	73.5	8.6	5272
	China	0.699	73.7	7.5	7945
	Turkmenistan	0.698	65.2	9.9	7782
	Thailand	0.69	74.3	6.6	7722
	*Maldives*	0.688	77.1	5.8	7478
	Mongolia	0.675	68.8	8.3	4245
	State of Palestine	0.67	73	8	3359
	Philippines	0.654	69	8.9	3752
	Uzbekistan	0.654	68.6	10	3201
	Syrian Arab Republic	0.648	76	5.7	4674
	Indonesia	0.629	69.8	5.8	4154
	Kyrgyzstan	0.622	68	9.3	2009
	Tajikistan	0.622	67.8	9.8	2119
	Viet Nam	0.617	75.4	5.5	2970
	Iraq	0.59	69.6	5.6	3557
	Timor-Leste	0.576	62.9	4.4	5446
	India	0.554	65.8	4.4	3285
	Cambodia	0.543	63.6	5.8	2095
	Lao PDR	0.543	67.8	4.6	2435
	Bhutan	0.538	67.6	2.3	5246
	Bangladesh	0.515	69.2	4.8	1785
	Pakistan	0.515	65.7	4.9	2566
Low	Myanmar	0.498	65.7	3.9	1 817
	Nepal	0.463	69.1	3.2	1137
	Yemen	0.458	65.9	5.3	928
	Afghanistan	0.374	49.1	3.1	1000
Unknown	*Korea, Democratic Republic of*	-	-	-	-

Abbreviations: GNI, gross national income (GNI); HDI, human development index.

### 4.1. ASIR and HDI

A positive correlation was seen between the ASIR of kidney cancer and HDI about 0.655. This association was statistically significant (*P*=0.001). There was a positive correlation between the ASIR and life expectancy at birth about 0.558 (*P*=0.001), positive correlation between the ASIR and mean years of schooling about 0.523 (*P*=0.001), and positive correlation between the level of income per each person of the population and the ASIR equal to 0.409 (*P*=0.005) (Figure 3).

In men, a positive correlation of 0.637 was observed between the ASIR of kidney cancer and HDI. It was statistically significant (*P*=0.001). There was a positive correlation between the ASIR and life expectancy at birth about 0.539 (*P*=0.001), positive correlation between mean years of schooling and the ASIR about 0.557 (*P*=0.001), and positive correlation between the level of income per each person of the population and the ASIR equal to 0.366 (*P*=0.012).

In women, a positive correlation of 0.612 was observed between the ASIR of kidney cancer and HDI. It was statistically significant (*P*=0.001). There was a positive correlation between the ASIR and life expectancy at birth about 0.509 (*P*=0.001), positive correlation between mean years of schooling and the ASIR about 0.448 (*P*=0.002), and positive correlation between the level of income per each person of the population and the ASIR equal to 0.347 (*P*=0.018).

### 4.2. ASMR and HDI

There was between the ASMR for kidney cancer and HDI a positive correlation of 0.285 (*P*=0.055), expectancy at birth a positive correlation of 0.183 (*P*=0.222), mean years of schooling a positive correlation equal to 0.226 (*P*=0.132), and the level of income per each person of population a positive correlation of 0.174 (*P*=0.248; Figure 3).

In men, there was between the ASMR for kidney cancer and HDI a positive correlation of 0.314 (*P*=0.033), expectancy at birth a positive correlation of 0.187 (*P*=0.212), mean years of schooling a positive correlation equal to 0.23 (*P*=0.029), and the level of income per each person of population a positive correlation of 0.152 (*P*=0.314).

In women, there was between the ASMR for kidney cancer and HDI a positive correlation of 0.131 (*P*=0.386), expectancy at birth a positive correlation of 0.076 (*P*=0.616), mean years of schooling a positive correlation equal to 0.045 (*P*=0.768), and the level of income per each person of population a negative correlation of 0.017 (*P*=0.913).

## 5. Discussion

Considering that close to 60% of the world’s population live in Asia, paying attention to causes of incidence and mortality from the cancer is significant in the continent (46). Lifestyle changes in Asian countries could be predisposing factor for the cancer (47,48). In Asia, 56% of incidence cases, 62% of deaths, 70% of DALYs occurred in 2013 worldwide. In Asian countries, ASIRs of incidence, mortality, and 5-year prevalence of kidney cancer in this year were 2.8, 1.3, and 9.4, respectively (1).

Kidney cancer incidence and mortality is different in various countries. This difference in incidence between countries is because of the accumulation of risk factors (49), including smoking, obesity, hypertension, age, and diet in countries with high incidence. According to studies conducted in Asian countries, age-standardized incidence rates per 100000 and the proportion of deaths to incidence per 100000 were in Central and South Asia 1 and 0.7, in Southeast Asia, 1.9 and 0.68, in West Asia 2.3 and 0.62, and in East Asia 2.4 and 0.36, while in the United States 11.8 and 2.2. Given the under-reporting in developing countries (50), the United States with a HDI has mortality rates a declining trend, but in Asian countries the rates are stable or increasing. Based the results of the this study and other studies, incidence and mortality rates of all cancers are different in the world and in various socioeconomic levels due to early detection, improved access to health care, complex diagnostic imaging, and treatment availability.

The incidence of kidney cancer has increased in many Asian countries, so that men, specifically in China, and Asian women in India have a significant increase. In a country like Singapore, trends in mortality have remained constant, but Japan has been a clear decline (46). In this study, the sex ratio (male to female) was 1.95, which is in line with other studies. This may be due to higher exposure to risk factors such as smoking and obesity (2,19,20,25,27,28,32).

According to the findings of this study, the incidence of kidney cancer in Asia is related to the HDI. It seems that among Asian countries, the high incidence of kidney cancer has been associated with increasing HDI. It can be attributed to a decrease in other diseases and control of infectious diseases as well as aging in the countries. Aging is one of the most important risk factors for this cancer. According to statistics published by GLOBOCAN 2015, the cancer generally has increased between 1990 and 2013. In other words, the incidence rate for both sexes in terms of ASIR increased 23% (from 3.82 to 4.7), 34% in developing countries (from 1.96 to 2.27) and 36 percent in developed countries (from 7.15 to 9.71) (1).

Our results showed that there was no relationship between the HDI and kidney cancer mortality rate in Asia. Of the characteristics of kidney cancer is no clinical signs warning, and RCC has been difficult malignancy in diagnose and treat. As a result, early diagnosis of the cancer is not different by the development in Asian countries.

Life expectancy at birth is one of components of human development. Our findings showed that there was a relationship between the ASIR and life expectancy at birth. Kidney cancer is a disease that is typically detected between the fifth and seventh life. In another study, incidence in Europe and the United States is constantly increasing, with a smooth trend in 70 to 75 years (32,51-53). Today, the average life expectancy for a child born in the United States is about 78 years, while a child in a country in Sub-Saharan Africa with an average life expectancy of between 39.6 to 65.9 years (33). Standard age distribution is various in different geographical areas. ASMR is similar to the ASIR, so that the highest in Europe and North America (3.1 and 2.6 per 100000) and the lowest in Asia and Africa (0.6 and 1.5 per 100000). With the increasing development of countries, aging of populations and reducing non-communicable diseases, rates of chronic disease such as cancer increasing. (54).

Access to knowledge is another component. Our study found a positive correlation between the standardized incidence and the level of education. Also, in United States, in the lowest level of education in the population, kidney cancer mortality rate is 2.6 times higher than the highest level of education (55). It was shown that risk of kidney cancer in men is inversely related to higher education levels (56).

In this study, there was a positive correlation between standardized incidence of the cancer and income levels per one in community. An ecological study has also reported that per capita daily intake of fat and protein is positively correlated with the incidence of kidney cancer in women and men (26). These findings are justified with aging (52) and an increase in cumulative effects of risk factors. The annual economic burden of kidney cancer in the United States in 2009 is estimated about $5.2 billion (43). About 85% of health care dollars is spent caring for kidney cancer inpatients (23). It can be concluded that the incidence of kidney cancer can also affect the HDI because it imposes economic costs on health systems and poverty in people (13).

## Conclusions

The ASIR and ASMR of kidney cancer in countries with higher development is more. There was a positive and significant relationship between the ASIR of kidney cancer and HDI and HDI components (life expectancy at birth, the average years of schooling, and the level of income for each one of the country’s population). The relationship is due to risk factors in countries with high development such as older age, smoking, hypertension, obesity, and diet. However there was a positive, but no significant relationship between the ASMR of kidney cancer and HDI and HDI components.

## Limitations of the study

Our study was an ecological study and special limitations of this study include ecological misleading and lack of relation of group results with individuals.

## Authors’ contribution

All authors contributed to the design of the research. AMH, MG, FD and FT collected the data. MG, MA and HS conducted analysis and interpretation of data. All authors drafted the first version. HS, KV and AMH edited the first draft. All authors reviewed and commented on final draft.

## Acknowledgements

Hereby we appreciate of the cooperation of all employees involved in data collection in the GLOBOCAN project and World Bank.

## Conflicts of interest

The authors declare no conflict of interests.

## Funding/Support

None.
